# Strangulation of the small intestine caused by an intra-mesosigmoid hernia: a case report

**DOI:** 10.1186/s40792-017-0406-z

**Published:** 2017-12-21

**Authors:** Kotaro Hirashima, Kazutoshi Date, Kanako Fujita, Norihiko Koide, Akihito Kamuro, Hiroshi Kato, Nobuhiro Fujita

**Affiliations:** 1 Department of Surgery, Joetsu General Hospital, 616 Daido-Fukuda, Joetsu City, Niigata 943-8507 Japan; 2 Department of Gastroenterology, Joetsu General Hospital, 616 Daido-Fukuda, Joetsu City, Niigata 943-8507 Japan; 3 Department of Radiology, Joetsu General Hospital, 616 Daido-Fukuda, Joetsu City, Niigata 943-8507 Japan

**Keywords:** Intra-mesosigmoid hernia, Strangulation, Internal hernia

## Abstract

Sigmoid mesocolon hernia is an uncommon type of internal hernia with only a few cases reported to date. This disease entity can progress rapidly to cause vascular disturbance, necrosis, and perforation of the bowel wall; therefore, early diagnosis and surgical treatment are essential. We describe the case of an intra-mesosigmoid hernia in a 60-year-old man without history of previous abdominal surgery who presented with sudden acute abdominal pain and vomiting. Based on computed tomography, which showed ascites and small bowel obstruction, we diagnosed him as having strangulation of the small intestine caused by a sigmoid mesocolic hernia and performed emergency surgery. Laparotomy revealed small intestinal strangulation, extensive engorgement, and discoloration of bowel loops. Approximately 100 cm of the small intestine extending from the ligament of Treitz had undergone strangulation and herniated into the defect of sigmoid mesocolon, leading to a diagnosis of an intra-mesosigmoid hernia. Because the incarcerated portion of the small intestine was viable, we did not perform intestinal resection and reconstruction but closed the defect in the sigmoid mesocolon. His postoperative course was uneventful.

## Background

Internal hernia is a protrusion of the small intestine or other abdominal organs through peritoneal or mesenteric orifices within the peritoneal cavity, occasionally leading to strangulation or incarceration. Additionally, an internal hernia is a rare cause of small bowel obstruction in patients without a history of abdominal surgery or trauma with a reported incidence of up to 5.8% of small bowel obstruction [1–3]. However, if strangulated and left untreated, internal hernias demonstrate a mortality > 50% [4, 5]. In cases presenting as an emergency, preoperative diagnosis is very difficult due to rarity of this entity and limited utility of imaging in cases of acute intestinal obstruction [6]. Sigmoid mesocolic hernia (SMH) is a rare type of congenital hernia, and intra-mesosigmoid hernia (IMSH) is the rarest among the SMHs [7–9]. We present a case of a rare type of primary internal hernia presenting with strangulation of the small intestine secondary to an IMSH in a 60-year-old man.

## Case presentation

**Fig. 1:**
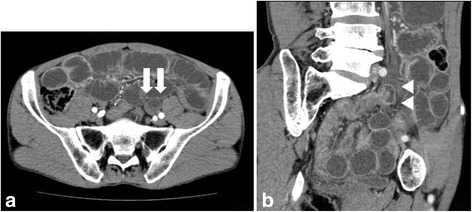
Abdominal computed tomography (CT) scan showing small *bowel obstruction*. **a** Axial section (white arrows). **b** Coronal section (white arrowheads)

A 60-year-old man with no history of similar episodes or any abdominal surgeries was admitted to our emergency department with sudden acute abdominal pain and vomiting. He showed no other symptoms except continuous vomiting and no flatus after onset. On general examination, pulse rate was 60/min, and blood pressure was 107/75 mm of Hg. He present abdominal distension, and bowel sounds decreased. Laboratory tests showed an elevated white blood cells (WBC) count (21.7 × 10^3^/μl) although his C-reactive protein (CRP) level was within reference range (Table 1). Computed tomography (CT) of the abdomen revealed small bowel obstruction with poor contrast effect (Fig. 1), and there appeared to be radial compression of the blood vessels of the sigmoid colon. The other organs appeared normal. We diagnosed strangulation of the small intestine secondary to SMH and performed an emergency laparotomy.

**Table 1:** Laboratory data on admission

WBC	21.7 × 10^3^/μl	Na	138 mEq/l
RBC	513 × 10^4^/μl	K	4.0 mEq/l
Hb	15.8 g/dl	Cl	99 mEq/l
Hct	46.8%		
Plt	16.3 × 10^4^/μl	BUN	19.9 mg/dl
		Cre	1.03 mg/dl
AST	19 U/l	CK	131 IU/l
ALT	18 U/l		
T-Bil	0.6 mg/dl	PT	92.0%
ALP	131 U/l	PT-INR	1.05
γ-GTP	34 U/l	APTT	97.8%
TP	8.2 g/dl		
Alb	4.67 g/dl		
T-Cho	218 mg/dl		
LDH	202 U/l		
CRP	0.32 mg/dl

**Fig. 2:**
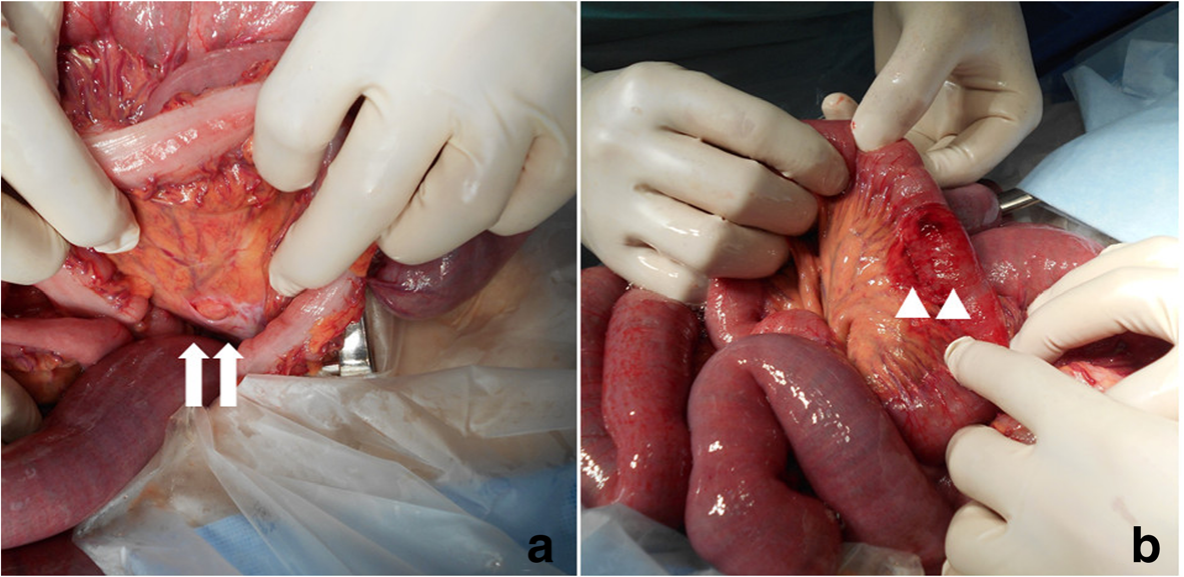
**a** Laparotomy showing a sigmoid mesocolon defect (white arrows). **b** Approximately 100 cm of the small intestine extending from the ligament of Treitz is seen to have herniated into the defect of sigmoid mesocolon (Richter-type hernia), although the incarcerated portion of the small intestine is observed to be viable (white arrowheads)

**Fig. 3:**
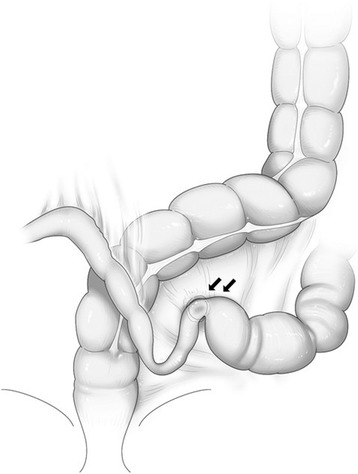
Schema showing detailed findings of the intra-mesosigmoid hernia in our case (black arrows)

Laparotomy revealed extensive engorgement of the small intestine and discoloration of bowel loops. Approximately 100 cm of the small intestine extending from the ligament of Treitz had been strangulated and showed herniation into the defect of sigmoid mesocolon (Richter-type hernia). The defect was incomplete and the right leaf of sigmoid mesocolon was remained, therefore, we diagnosed as an IMSH (Figs. 2 and 3). Because the incarcerated portion of the small intestine was viable, we did not perform intestinal resection and reconstruction, but we closed the defect in the sigmoid mesocolon. His postoperative course was uneventful, and he started to take a meal on the 3rd postoperative day and was discharged from our hospital on the 6th postoperative day.

### Discussion

Internal hernia is caused by defects within the peritoneal cavity, which might be congenital, postoperative, and/or idiopathic. The incidence of internal hernias based on autopsy diagnosis is 0.2–2.0%, and most of them are asymptomatic [1]. SMH (a type of internal hernia) is uncommon, accounting for approximately 5% of all internal hernias [10, 11].

Benson et al. have classified SMHs into three types [10]: (1) intersigmoid hernia (ISH): herniation into the intersigmoid fossa, situated at the attachment of the lateral aspect of the sigmoid mesocolon. This fossa is formed during fusion of the left peritoneal surface of the sigmoid mesentery with the parietal peritoneum of the posterior abdominal wall, the line of Toldt. (2) Trans-mesosigmoid hernia (TMSH): incarceration of intestinal loops through an isolated oval defect in the sigmoid mesocolon. No hernial sac is present. (3) Intra-mesosigmoid hernia (IMSH): a congenital, oval defect unrelated to the intersigmoid fossa is present in juxtaposition to the colon and involves only one leaf (lateral more common) of the sigmoid mesocolon with herniation. Normal fusion fascia is present, and the right leaf is intact in this setting. Our case is classified as an IMSH, which is the rarest type based on Benson’s classification.

CT plays an important role in the evaluation of intestinal obstruction and acute abdomen [2]. It is a valuable tool for early diagnosis and planning for surgical treatment in patients with internal hernia. However, preoperative diagnosis of internal hernia using CT remains very difficult even after the present when it spread widely. Ours was a case of SMH, for which in a majority of cases, diagnosis can be confirmed only during surgery [12, 13]. However, a radiologist at our hospital could preoperatively confirm diagnosis of SMH in our case. Our patient related no history of abdominal surgeries or trauma. However, patients presenting with small bowel obstruction and a positive history of tuberculosis or previous surgeries should be evaluated for the possibility of an internal herniation causing the obstruction.

Patients with small bowel obstruction refractory to conservative therapy require emergency surgical treatment if an internal herniation is suspected, and laparoscopy is useful if there is no evidence of strangulation or necrosis. Recently, some reports have recommended laparoscopic abdominal surgery for both diagnosis and surgical treatment [11, 14, 15]. Cases presenting with an ISH and IMSH do not show a complete defect in the mesosigmoid; therefore, laparoscopic detachment of peritoneal adhesions to enable sigmoid colon mobilization is often possible and sufficient. Moreover, the defect can be sutured laparoscopically. In our case, we performed an open laparotomy because the small intestine was filled with intestinal fluids and grossly engorged due to intestinal obstruction.

## Conclusions

We describe a case of an IMSH, which is a rare type of SMH. Although rare and often underdiagnosed, internal hernias are an important cause of intestinal obstruction given the high mortality associated with this disease entity. Primary internal hernias should be considered among the differential diagnosis in adults with no previous history of surgery or trauma who present with acute intestinal obstruction. If clinicians identify an internal hernia, it may be better to take SMH into consideration as a likely diagnosis, which ought to be immediately confirmed followed by emergency surgical treatment.

## Abbreviations

CRP: C-reactive protein
CT: Computed tomography
IMSH: Intra-mesosigmoid hernia
ISH: Intersigmoid hernia
SMH: Sigmoid mesocolic hernia
TMSH: Trans-mesosigmoid hernia
WBC: White blood cells
